# Prevention of Opioid Misuse: A Summary with Suggestions from a Pain Working Group

**DOI:** 10.1155/2016/8708654

**Published:** 2016-04-07

**Authors:** Gilles J. Lavigne

**Affiliations:** Faculty of Dental Medicine, Université de Montréal and Surgery Department, Hôpital du Sacré-Coeur de Montréal, CP 6128, Succursale Centre Ville, Montreal, QC, Canada H3C 3J7

## 1. Introduction

On November 6, 2014, a multidisciplinary group of experts and stakeholders attended a Prevention of Opioid Misuse Study Day in Toronto funded by the Canadian Institutes of Health Research (CIHR) under a Canada Research Initiative in Substance Misuse (CRISM, grant number CSM-133338).

The* goals* were to

1. clarify issues surrounding opioid medications, including a discussion of current facts and myths,
2. produce a summary of evidence based information to guide health professionals on the best practices in managing acute and chronic pain,
3. inform clinicians, government, and other stakeholders about opioid use/misuse and pain management issues.

*Specific Principles That Guided the Summary.* The following are the specific principles that guided the summary:

1. We recognize the rise in morbidity and mortality associated with opioid misuse and the risk of addiction.
2. We recognize the importance of the International Association for Study of Pain, Montréal, 2010 declaration on the right of person with pain to get relief: http://www.iasp-pain.org/DeclarationofMontreal.
3. We recognize that available alternatives to opioids should be used in first intention (e.g., nonsteroidal analgesics, pregabalin, mood related medication such as duloxetine, and physical and behavioral approaches).
4. We endorse the 2010 Canadian Guideline for Safe and Effective Use of Opioids for Chronic Non-Cancer Pain: http://nationalpaincentre.mcmaster.ca/opioid/. These are solid and remain valid.
5. We also deplore the low access to pain experts and other management options in Canada (mainly in rural areas or low income urban ones) and the absence of newer medications with high effectiveness and low harm.

## 2. Review of Current Issues on Pain and Its Relief

One in five Canadians suffers from chronic pain [1, 2]. Many people living with chronic pain are “invisible” sufferers. Their quality of life is seriously compromised, and if their pain is not managed they are at greater risk of additional problems. Importantly, an association between opioid medication misuse and mood problems including depression and anxiety has been reported [3]. Furthermore, in the absence of adequate pain relief, the risk for suicide increases [4, 5].

Unfortunately, there are no magic cures for chronic pain. When all other alternatives have failed to provide acceptable control of pain, opioid analgesics (e.g., morphine, oxycodone, hydromorphone, and fentanyl) are increasingly being prescribed to relieve pain and improve the quality of life for people living with chronic pain so they can continue working and carrying out routine daily tasks. However, opioids if used inappropriately come with definite serious risks: addiction, fatal overdose, and harmful or even deadly effects when combined with other drugs or substances (particularly alcohol and antianxiety medications). The prevention of opioid misuse requires careful screening by educated prescribers, increased safety education to patients and population, and increased access to addiction and mental health services across all age groups and regions in Canada with a special attention to access to pain management and its prevention in nonurban sites [6].

It is important to distinguish between abuse, addiction, and physical dependence (see page 124 of the Canadian Guidelines: http://nationalpaincentre.mcmaster.ca/documents/opioid_guideline_part_b_v5_6.pdf):

- *Abuse* is any use of an illegal drug or the intentional self-administration of a medication for a nonmedical purpose such as altering one's state of consciousness, for example, “getting high.”
- *Addiction* is a primary, chronic, neurobiological disease with genetic, psychosocial, and environmental factors influencing its development and manifestations. It is characterized by behaviors that include one or more of the following: impaired control over drug use, compulsive use, continued use despite harm, and craving.
- *Misuse of opioid* is use of an opioid in ways other than those intended by a prescribing health professional.
- *Physical dependence* is a state of adaptation manifested by a drug class-specific withdrawal syndrome that can be produced by abrupt cessation, rapid dose reduction, decreasing blood level of the drug, and/or administration of an antagonist.

The nonmedical use of any prescription medication, including opioids, is a major concern. All medications have adverse effects, but if opioids are inappropriately used they are potentially life-threatening due primarily to sedation and respiratory depression. It is important to address this problem and to implement appropriate solutions. The national Canadian Alcohol and Drug Use Monitoring Survey (CADUMS) of the general population aged 15 years and older included nonmedical users who acknowledged using pain relievers more than they were supposed to or without a prescription. Starting in 2009, CADUMS has also asked respondents if they had used the medication “to get high.” The findings indicated that the prevalence of any use of a prescription opioid analgesic (POA) in Canada had decreased from roughly 20% to 17%, with use without a prescription at about 4.8–5% and use to get high at about 0.3%.

We need to know more about the 0.3% of opioids' users identified by CADUMS who are using them to get high. Knowing more about this population would help us to find ways to prevent them from using dangerous drugs to get high. What about the other roughly 4.7% of the 5% who are using POAs either more than they should or without a prescription? Why are they doing this? Is it to treat their pain or is it due to some form of oligoanalgesia (i.e., lack of adequate pain relief was observed in emergency in close to 40% of patients) and why do strong opioids remain the most frequently prescribed [7, 8]?

The bottom line is that we do not have a clear answer to this problem. However, the literature can shed some light on this issue. Several studies have examined motivation for Nonmedical Prescription Opioid Use (NMPOU) and found that the single leading reason was for relief of pain [9–11]. Pain patient groups are very sensitive about the stigmatization that some opioid users for chronic pain have to face. Many health professionals are uncomfortable managing patients on opioids for long periods [12]. Education is needed to reduce misuse risk in parallel with improving patient's pain management and prevent opioid stigma to people needing opioid.

Opioid misuse remains a growing public health problem and a dilemma for clinicians and people with chronic pain [13]. In a 2008 systematic review of studies examining the risk of developing abuse, addiction, or other aberrant drug-related behaviors in patients exposed to chronic opioid therapy for chronic pain, it was found that the incidence of opioid abuse or addiction after chronic opioid exposure was low, at 3.27%, and, in studies of patients with no previous history of abuse or addiction, the incidence was even lower, at 0.19% [14]. In a 2015 systematic review and data synthesis of 38 published studies, the rate of opioid misuse is reported to range between 21 and 29% and for addiction between 8 and 12% [15]. All these numbers reveal that it is a serious problem; however, a few clarifications are needed. In acute use, it is 0.12% of opioid users that developed opioid use disorders, that is, abuse or dependence; in the chronic user it is 6.1% and this was dependent on dose and duration of use (more than 91 days) [16].

It is obvious that we have to address both misuse risk and improvement of undertreated pain in Canada due in part to a critical lack of access to pain treatments (nonpharmacological and pharmacological), most of which do not involve opioids and many of which are not drugs at all but involve working with allied health professionals and interdisciplinary pain management teams [17].

Another not fully understood critical issue emerging after the introduction of tamper-resistant opioid analgesics (oxycodone) is the rise in heroin use [18–20]. Diversion of opioid misuse to heroin in college students needs to be better understood; it is a no-brainer that education on such harm is needed [19, 21]. Other illicit substances are also used. Such substitutions may reflect a shift in the public health problem to other deleterious products (see Anna Mehler Paperny's “UPDATED: Opioids Killing More Ontarians Than Ever, Coroner's Numbers Show,”* November 9, 2014, available at* http://globalnews.ca/news/1625100/opioids-killing-moreontarians-than-ever-coroners-numbers-show/).

Again, the overriding challenge for the healthcare system is to find a balance between reducing the harm associated with the nonmedical use/misuse of opioids and providing people experiencing both acute and chronic pain with access to optimal treatments. Combinations of physical, psychological, and pharmacological interventions are needed, with stronger pain medication such as opioids for more severe pain.

Another challenge is the lack of specific information in available guidelines to indicate the subpopulation of chronic pain patients who are most likely to benefit from nonopioid and opioid treatment with the minimal risk of addiction or misuse (work is in progress on that matter). More work is then needed to find the best algorithm for management of acute pain (e.g., with procedures, surgery, or trauma) to prevent chronic pain. It is noteworthy that the new International Classification of Disease 11 for chronic pain developed by experts from the International Association for the Study of Pain includes (1) chronic primary pain, (2) chronic cancer pain, (3) chronic posttraumatic and postsurgical pain, (4) chronic neuropathic pain, (5) chronic headache and orofacial pain, (6) chronic visceral pain, and (7) chronic musculoskeletal pain [22]. No simple and unique approach will be effective in managing all pain types. The search continues for the most effective pain relief methods with the least harm, that is, fewer adverse effects and the lowest addiction risk. Approaches need to be tailored for each type of pain.

An obvious important strategy is to increase education for primary healthcare providers who have a poor understanding of the best practices for pain management including the appropriate use of medications [12]. Patients and families also need education about the safe and appropriate use of analgesics, including opioids.

In addition, greater collaboration is required between pain and addiction specialists. More mental health resources are needed to allow early treatment of addiction issues when recognized. There is a need to involve all of our partners (i.e., the public, patients, universities, and governments) in raising awareness about appropriate and inappropriate use of drugs, including opioids. Broader education is required of the public and governments as well about the need for further research about appropriate pain treatments, increased pain education of health professionals, and more access to knowledgeable health professionals for people who need ongoing pain management. A national strategy on these issues is needed and Health Canada has made a positive move into that direction with the CRISM in partnership with CIHR, as well as other initiatives; see http://news.gc.ca/web/article-en.do?nid=969739.

## 3. The Group Proposed a Road Map with Three Main Avenues for Action to Reduce Opioid Misuse/Addiction: (1) Access to Pain Management and Better Education to Population and Health Professionals, (2) Knowledge Transfer Strategy, and (3) Applied Research for Innovation in Pain and Addiction Prevention

The following section summarizes the exchanges between patient group representatives, public health experts, pain clinicians, and researchers who participated in the Study Day.

### 3.1. Avenue 1: Access to Pain Management and Better Education to Population and Health Professionals

*Goals*. Goals are to be more knowledgeable about clinical and community strategies to prevent persistent pain and/or medication misuse.

The group strongly encourages increasing the

- access to nonpharmacological and to nonopioid pharmacological treatments at first,
- content on pain assessment and management in healthcare education curricula, including the use and monitoring of opioid analgesics,
- content of continuing education on pain treatments, including nonopioid and opioid analgesics approaches and misuse/addiction prevention, to all healthcare practitioners.

*Paths*. Consider the following:

- Greater access to the nonpharmacological pain management methods is needed. This includes nonurban sites and specific populations with chronic pain.
- It is important to integrate new knowledge and the best practices into competency-based training programs for healthcare professionals (e.g., nurses, physicians, pharmacists, dentists, psychologists, psychiatrists, physical therapists, occupational therapists, and social workers), other related professionals (e.g., addiction counsellors, teachers, researchers, and law enforcers), and private stakeholders (e.g., pharma and insurance companies).
- Education is needed on optimal pain treatments, including pharmacological, physical, and psychological strategies. Treating acute and chronic pain should begin with nonopioids (e.g., combination of ibuprofen and acetaminophen) [23] (or short-action duration opioids) and, as appropriate, other nonpharmacological strategies (e.g., relaxation exercises, physical therapy, and sleep hygiene).
- The available screening tools and risk assessment algorithms need to be disseminated and used in practice to help healthcare professionals detect and prevent opioid misuse. These screening tools cover any addiction history, mental health problems (especially depression and anxiety), sleep disorders (especially insomnia), socioeconomic problems, and drugs used for comorbidities (e.g., hypertension, obesity, diabetes, and inflammatory diseases).
- Education is also needed on how to monitor ongoing opioid treatments so that all members of the pain treatment team are aware of medication use duration, potential drug misuse, multiple prescriptions, and the effects of comorbidities and other drugs on patient outcomes. Implementation of an electronic prescription drug database would allow monitoring of drug use by healthcare workers but is presently available in only a minority of provinces.
- Once medication misuse or illicit drug use is suspected, a strategy should be developed (with community input) to have access to addiction specialists or other qualified professionals. Although pain and addiction are being comanaged in Canada, this strategy is still in its infancy and needs to be promoted.

The overall outcome would be more integrated pain management across primary healthcare providers, pain clinics, and addiction/mental health treatment centres.

### 3.2. Avenue 2: Knowledge Transfer (KT) Strategy

*Goals*. The group recognizes that greater awareness and understanding by governments, the media, industry, and the general public of the issues surrounding the appropriate use and misuse of opioid analgesics would be best achieved through multimedia exposure.

*Paths*. Consider the following:

- It is critical to raise awareness and disseminate accurate information about chronic pain and the importance of timely and appropriate treatment. The media and the Internet offer effective vehicles for such messages, including personal stories from people with pain to help balance concerns about opioid use, misuse, and abuse.
- Greater coordination as well as knowledge sharing is needed within and across provinces.
- It is vital for new findings on pain and pain management to be disseminated in journals such as Pain Research and Management, at conferences like the Canadian Pain Society Annual Scientific Meeting and other Canadian health professional journals and meetings.
- It is also critical to support efforts to raise awareness about the appropriate screening for and management of substance dependency and addiction.
- It is critical that all stakeholders, regulatory agencies, and pain and addiction groups work together to have a high impact strategy and avoid silo packaging in message to the public and health professionals.

### 3.3. Avenue 3: Applied Research for Innovation in Pain and Addiction Prevention

*Goals*. The group identified the need that more research was necessary to determine the relationship between the use of opioid analgesics for persistent pain, mental health, and addiction risk. A balance is needed between ensuring pain relief for those needing opioids and the potential for opioid misuse or abuse.

*Paths*. Consider the following:

- Research methodologies should be rational, collaborative, and interdisciplinary (including inputs from communities, patients, law enforcers, and pain and addiction professionals).
- Innovative research methodologies with a stakeholder approach should be developed, and alternative treatments, including nonpharmaceutical methods and devices, should be assessed as appropriate.
- Newer modalities with less adverse effects need to be developed.

## 4. Many Questions Remain Unanswered

Examples of these questions are as follows. Preventing risk as well as consequences of misuse and addiction/right for pain relief is as follows:

- What are the barriers to better access to pain management, misuse prevention, and addiction treatment for all ages and regions in Canada?
- What are the best, evidence based, addiction screening tools?
- Which tools can be easily used in the various treatment contexts of medicine, surgery, and emergency or ongoing persistent pain management?
- How can we improve nonopioid versus opioid use guidelines and efficient management practices?
- How can we overcome the various barriers to use of guidelines on pain management practices and on misuse/prevention strategies (e.g., time, risks, law enforcement policies, and protection of people requiring analgesics) [12]?
- How can we prevent or reduce the social stigmatization of people who have to take prescribed opioids to maintain their quality of life and acceptable pain relief?
- How can we reduce the number of opioid prescriptions without inducing harm, that is, diversion to other medications or illicit products to get pain relief?
- How can we reach population at risk of diversion or opioid misuse to prevent serious health consequence and death (e.g., college students, chronic pain users, and mental health patients)?
- What are the motives and dynamics behind drug use, misuse, and addiction (e.g., high-risk dosages, early-use risks, precipitating factors, genetic factors, and environmental influences)?

*Mechanism of Prevention and Pain Management Challenges.* Consider the following:   

- Studies on reward mechanisms would be a valuable research avenue in pain management and misuse or addiction prevention.
- How can the role of comorbidities (e.g., anxiety, depression, insomnia, and other compulsive behaviors) be disentangled?
- What are the long-term benefit and risk of various pain treatments and pain management strategies for acute and chronic pain?
- What are the causal mechanisms of pain chronicity, NMPOU behaviors, addiction, and mortality?
- Can optimal treatment strategies and durations be determined for a variety of chronic pain conditions, including neuropathic pain, musculoskeletal pain, and visceral pain? The* one-size-fits-all* approach is inadequate for a patient-centred research paradigm.

## Additional Points

Participant profiles are as follows: Aline Boulanger, M.D., F.R.C.P.C., M.P.H., is Director of Pain Clinic, Centre Hospitalier de l'Université de Montréal (CHUM). Norman Buckley, M.D., F.R.C.P.C., is Chair and Professor at Department of Anesthesia and Director of McMaster University Michael G. DeGroote National Pain Centre. Brian E. Cairns, Ph.D., is Professor at Faculty of Pharmaceutical Sciences, University of British Columbia, and President-Elect of Canadian Pain Society. Manon Choinière, Ph.D., is Senior Pain Researcher, Department of Anesthesiology, Université de Montréal and CHUM. Hance Clarke, M.D., Ph.D., F.R.C.P.C., is Director of Transitional Pain Program; Medical Director of Pain Research Unit and Staff Anesthesiologist, Toronto General Hospital; and Assistant Professor at University of Toronto. Lynn Cooper is President of Canadian Pain Coalition for persons who live with pain. Bob Forbes, B.A., M.A.Ed., is National Medical Education Manager of Johnson & Johnson Inc. Louis Gendron, Ph.D., is Associate Professor at Department of Physiology and Biophysics, Université de Sherbrooke. Jacques Laliberté is President of Association Québécoise de la Douleur Chronique (AQDC), for sustained improvement in pain prevention and management. Gilles Lavigne, D.M.D., Ph.D., F.R.C.D., is Professor and Dean, Canada Research Chair in Pain, Sleep and Trauma, Faculty of Dental Medicine, Université de Montréal, and Surgery Department, Hôpital du Sacré-Coeur de Montréal, and President of Canadian Pain Society. Mary Lynch, M.D., F.R.C.P.C., is Professor of psychiatry, anesthesia, and pharmacology, Dalhousie University, Halifax, and Past President of Canadian Pain Society. Margaret McKyes is Certified Translator, Certified Teacher, Scientific Writer, and Member of OTTIAQ, EAC, and IFT. Dwight Moulin, M.D., is Professor at Departments of Clinical Neurological Sciences and Oncology, Western University, and Earl Russell Chair of the Western Pain Program, Western University, London, Ontario. Rita Notarandrea is Interim Chief Executive Officer (CEO), Canadian Centre on Substance Abuse, Ottawa. Grant Perry is *‎*Vice President of Corporate and Government Affairs at Purdue Pharma, Canada. Paula Robeson, B.N., M.S.N., Team Leader of Knowledge Mobilization, Canadian Centre on Substance Abuse, Ottawa. Judy Watt-Watson, RN, M.S., Ph.D., is Professor Emeritus, Lawrence S. Bloomberg Faculty of Nursing, Senior Fellow, Massey College, University of Toronto, and Immediate Past President, Canadian Pain Society.
